# Blood borne: bacterial components in mother’s blood influence fetal development

**DOI:** 10.14800/ics.1421

**Published:** 2016

**Authors:** Allister J. Loughran, Elaine I. Tuomanen

**Affiliations:** Department of Infectious Diseases, St. Jude Children’s Research Hospital, Memphis, TN, 38105, USA

**Keywords:** Fetal development, bacterial cell wall, maternal fetal interface, neuroproliferation, fetal TLR2 response, autism

## Abstract

Bacterial or viral infection of the mother during the course of pregnancy can cross the placenta and actively infect the fetus. However, especially for bacteria, it is more common for mothers to experience an infection that can be treated without overt fetal infection. In this setting, it is less well understood what the risk to fetal development is, particularly in terms of neurological development. This research highlight reviews recent findings indicating that bacterial components generated during infection of the mother can cross the placenta and activate the fetal innate immune system resulting in changes in the course of brain development and subsequent progression to postnatal cognitive disorders. Bacterial cell wall is a ubiquitous bacterial PAMP (pathogen-associated molecular pattern) known to activate inflammation through the stimulation of TLR2. Cell wall is released from bacteria during antibiotic treatment and new work shows that embryos exposed to cell wall from the mother demonstrate anomalous proliferation of neuronal precursor cells in a TLR2 dependent manner. Such proliferation increases the neuronal density of the cortical plate and alters brain architecture. Although there is no fetal death, subsequent cognitive development is significantly impaired. This model system suggests that bacterial infection of the mother and its treatment can impact fetal brain development and requires greater understanding to potentially eliminate a risk factor for cognitive disorders such as autism.

## Introduction

During pregnancy, infection and inflammation can pose a threat to the health and development of the fetus ^[3, 14, 24]^. While the ability of a number of bacterial and viral pathogens to cross the placental barrier and cause harm to the development of the fetus is known, less well studied is the impact that bacterial components tracking from the maternal blood stream to the fetus have on development. Microbial products circulate not only in active infection but also normally when released from the gut microbiome ^[5]^. In this emerging field, new evidence suggests the mother’s intestinal microbiota during pregnancy influences early innate immune responses in her offspring. Gomez de Aguero *et al* found that specific bacterial metabolites from the mother’s microbiome induce the development of the ILC3-IL-22 antimicrobial system in the fetus ^[10]^. This was the first report definitively showing the role of the mother’s microbiome on fetal development in utero. Further evidence suggests that changes in maternal cytokine profiles, gut microbial dysbiosis and even bacterial metabolites and fragments crossing the placenta induce fetal responses ^[4, 10, 11]^. Not all interactions with bacterial products at the maternal-fetal interface are positive. This research highlight focuses on the ability of cell wall, a universal component of bacteria, to cross the placenta and disrupt the normal development of the murine fetal brain, resulting in cognitive disorders after birth ^[11]^.

Pathogen associated molecular patterns (PAMPs) are recognized by the innate immune system as an early sign of infection. In the case of the Gram positive pathogen *Streptococcus pneumoniae,* a major PAMP is the cell wall (CW) which triggers innate immune responses through toll like receptor 2 (TLR2) ^[12, 27]^. *S. pneumoniae* is a leading cause of infant and adult bacterial sepsis and meningitis, a serious infection leading to significant brain damage even when treated. In animal models, bacterial CW is sufficient to induce the entire symptom complex of meningitis ^[25, 26]^. It can traffic across the blood brain barrier and trigger inflammation and neuronal death ^[2, 8, 20]^. Much like the blood brain barrier, the placenta acts as a physical barrier between the mother and the immune privileged embryo, thereby protecting against invading pathogens and the mother’s immune system. Pathogens that are able to infect the fetus and disrupt development share the ability to cross the placental barrier ^[22]^. However, the ability of bacterial components to cross the placenta has not been well studied. Given that CW fragments can cross the blood brain barrier in postnatal models, we reasoned that CW exposure to pregnant mice would be a good model to assess the impact of sterile bacterial products on the developing fetal brain.

## CW silently crosses the placenta and fetal blood brain barrier

To model the circulation of bacterial products during infection, FITC labelled CW was injected intravenously into pregnant dams and followed by microscopy for accumulation in the placenta and fetal brain. CW crossed the placenta without significant inflammation, cell death or placental dysfunction ^[11]^. Within 24 hours, CW accumulated in the fetal brain. The translocation process across the placenta and the fetal blood brain barrier was mediated by the platelet activating factor receptor (PAFr). PAFr binds the chemokine PAF but by molecular mimicry also binds and transports components of most respiratory pathogens that decorate their surface with phosphorylcholine ^[6]^. This implies that components of many bacteria can easily cross to the developing fetus.

It is well known that CW causes inflammation and cell death in postnatal animal models ^[2, 8, 20, 25]^. Surprisingly, the accumulation of the CW in the fetal brain elicited very little neuronal death or influx of inflammatory cells. Rather the fetal brain responded by proliferation of neuronal precursor cells (NPCs) leading to a 50% increase in the density of cells at the cortical plate ^[11]^. NPC proliferation in response to CW was recapitulated *in vitro* using primary murine NPCs. This phenotype both *in vitro* and *in vivo* was TLR2 dependent as transgenic mice lacking TLR2 did not show fetal neuroproliferation.

## The mechanism of CW induced neuroproliferation

Neuroproliferation of NPCs appears to involve activation of the neuronal transcription factor FoxG1. Forkhead box protein G1 (FoxG1) is important in early development ^[16]^ and is embryonic lethal when knocked out ^[9]^. Mutations in the *FOXG1* gene are the cause of *FOXG1* syndrome, a condition consisting of developmental and structural brain abnormalities ^[13]^. In pre-natal brains, exposure to CW led to a significant increase in FoxG1 expression in a TLR2 dependent manner ^[11]^. This indicates a previously unknown link may exist between innate immunity (TLR2 activation) and neurodevelopment (FoxG1 expression).

Using the *in vitro* model of NPC proliferation induced by CW, distinction could be made as to activation of the partners for TLR2: e.g. heterodimer TLR2/6 or TLR1/2 ^[11]^. Synthetic agonists of TLR2/6 but not TLR2/1 caused neuroproliferation suggesting that CW induced signaling may be restricted to a specific arm of the classical innate immune response pathway. Currently both TLR2 heterodimers are thought to signal through the adapter molecule MyD88 and end in NF-κB activation leading to the release of inflammatory cytokines ^[19]^. If both receptors do in fact signal through the same pathway, it suggests that a second signal or novel pathway may exist to change the outcome from neuronal death with inflammation to neuroproliferation. The literature on the TLR1/2 complex is far more complete than that of the TLR2/6 heterodimer suggesting this may be a good model for further elucidation of the TLR2/6 signaling cascade. Of particular interest in this model is the role of PI3K-AKT to the neuroproliferation pathway. During neuroproliferation there was an increase in p-AKT and PI3Kinase both of which are in the FoxG1 signaling cascade ^[23]^. Furthermore hyperactivation of the PI3K-AKT pathway leads to a mislocation of neurons through a pathway involving FoxG1 ^[1]^. Mislocation of neurons is not unlike the phenotype observed in response to CW exposure indicating a possible relationship between increased PI3K-AKT and the FoxG1 overexpression phenotype.

In the brain, immune surveillance is carried out by the resident macrophages, the microglial cells ^[21]^. Microglial activation marker, Iba-1, which is strongly upregulated in the adult brain in response to CW, was decreased in the CW exposed fetal brain. This suggests that the CW either does not activate microglia in the fetal brain or leads to loss of microglia. The microglia play an important role during neural development in regulating the NPC population density ^[7]^. Potential loss or lack of activation of microglia in response to CW exposure may contribute to the proliferation of the NPCs in the fetal brain; however this remains to be determined.

## Postnatal cognitive impairment

The prenatal neuroproliferation and increased cortical plate cell density induced by CW did not result in fetal death or apparent abnormalities at birth. However, when examined over time after birth, pups exposed to CW gained less weight and at 3 months of age showed significant behavioral abnormalities ^[11]^. Pups showed less exploration, impaired memory, and more isolation. These abnormalities showed a time dependence in that only CW exposure between E10 and E15 resulted in abnormal brain architecture and cognitive impairment. Given that genetic studies show a link between increased expression of FoxG1 and autism ^[15]^, and given disruption of microglial development has also been linked to autism ^[17]^, further studies to define the spectrum of CW induced abnormal behaviors is warranted.

## Clinical implications

Bacterial products circulating in the blood stream are released in an explosive manner when bacteria are killed by CW-active antibiotics, such as the β lactams. ^[18]^. In animal models and human infection, treatment with β lactams such as ampicillin is accompanied by a burst of inflammation as a result of abrupt release of bacterial products into the surrounding tissues or the bloodstream ^[25, 26]^. We reasoned that CW released during antibiotic treatment of maternal infection may also lead to abnormal fetal brain architecture. When pregnant dams were infected with living *S. pneumoniae* and cured by treatment daily for five days with ampicillin, embryos showed the predicted significant increase in cell density at the cortical plate as observed when challenged with purified CW ^[11]^. Importantly when the infected mother was treated with clindamycin, a protein synthesis inhibitor that does not release CW, there was no increase in the cortical plate cell density. The findings of this study could have important clinical implications for the choice of antibiotics for treatment of suspected infection during pregnancy.

## Future studies

Given the emerging findings of the effect of microbial products on fetal development, this will be an important area for future studies of pathophysiology at maternal fetal interface. Questions raised include the mechanisms of alteration of fetal development by activation of innate immune signaling. The interacting pathways appear to crosstalk differently over the course of pregnancy and to switch completely around the time of birth. The details of the differential effect of CW on neurons pre- and postnatally suggest a route to potentially develop novel therapeutics to block the inflammatory pathway observed in the postnatal setting and encourage new neuronal development to mitigate the devastating effects of meningitis. On the other hand, preventing neuroproliferation in the fetus may lower the incidence of postnatal developmental disorders. Epidemiological studies support a link between fever, FoxG1 and autism spectrum which may be further explored by this model ^[3, 14]^. Questions about CW release from antibiotic treatment of maternal infection or the microbiome may lead to new therapeutic guidelines.

## Summary

The study of Humann *et al* ^[11]^ highlights that bacterial subcomponents and not just living bacteria can pose a threat to the fetus during gestation. There is a need to take into account the effects of bacterial metabolites and byproducts in the blood during pregnancy on fetal development. Therefore, consideration of how to treat maternal infection may need to include an assessment of how components released during death of the infecting organism impacts the developing fetus. This model revealed the role of innate immune signaling by TLR2 during embryogenesis and the potential to modulate responses to prevent both abnormal neuroproliferation and neuronal death in response to bacterial products. This study focused on the TLR2 PAMP, bacterial CW, but the release of bacterial products into the maternal bloodstream from other species of bacteria may also effect fetal development. This emerging field will foster new understanding in fetal brain development, fetal immune development, interactions between PAMPs and neuronal tissues and cell signaling pathways.

## References

[1] Baek ST, Copeland B, Yun EJ, Kwon SK, Guemez-Gamboa A, Schaffer AE (2015). An AKT3-FOXG1-reelin network underlies defective migration in human focal malformations of cortical development. Nat Med.

[2] Braun JS, Novak R, Herzog KH, Bodner SM, Cleveland JL, EI, Tuomanen EI (1999). Neuroprotection by a caspase inhibitor in acute bacterial meningitis. Nat Med.

[3] Canetta S, Sourander A, Surcel HM, Hinkka-Yli-Salomaki S, Leiviska J, Kellendonk C (2014). Elevated maternal C-reactive protein and increased risk of schizophrenia in a national birth cohort. Am J Psychiat.

[4] Choi GB, Yim YS, Wong H, Kim S, Kim H, Kim SV (2016). The maternal interleukin-17a pathway in mice promotes autism-like phenotypes in offspring. Science.

[5] Clarke TB, Davis KM, Lysenko ES, Zhou AY, Yu Y, Weiser JN (2010). Recognition of peptidoglycan from the microbiota by Nod1 enhances systemic innate immunity. Nat Med.

[6] Cundell DR, Gerard NP, Gerard C, Idanpaan-Heikkila I, Tuomanen EI (1995). Streptococcus pneumoniae anchor to activated human cells by the receptor for platelet-activating factor. Nature.

[7] Cunningham CL, Martinez-Cerdeno V, Noctor SC (2013). Microglia regulate the number of neural precursor cells in the developing cerebral cortex. J Neurosci.

[8] Fillon S, Soulis K, Rajasekaran S, Benedict-Hamilton H, Radin JN, Orihuela CJ (2006). Platelet-activating factor receptor and innate immunity: uptake of gram-positive bacterial cell wall into host cells and cell-specific pathophysiology. J Immunol.

[9] Frullanti E, Amabile S, Lolli MG, Bartolini A, Livide G, Landucci E (2016). Altered expression of neuropeptides in FoxG1-null heterozygous mutant mice. Eur J Hum Genet.

[10] Gomez de Aguero M, Ganal-Vonarburg SC, Fuhrer T, Rupp S, Uchimura Y, Li h (2016). The maternal microbiota drives early postnatal innate immune development. Science.

[11] Humann J, Mann B, Gao G, Moresco P, Ramahi J, Loh LN (2016). Bacterial Peptidoglycan traverses the placenta to induce fetal neuroproliferation and aberrant postnatal behavior. Cell Host Microbe.

[12] Iwaki D, Mitsuzawa H, Murakami S, Sano H, Konishi M, Akino T (2002). The extracellular toll-like receptor 2 domain directly binds peptidoglycan derived from Staphylococcus aureus. J Biol Chem.

[13] Kortum F, Das S, Flindt M, Morris-Rosendahl DJ, Stefanova I, Goldstein A (2011). The core FOXG1 syndrome phenotype consists of postnatal microcephaly, severe mental retardation, absent language, dyskinesia, and corpus callosum hypogenesis. J Med Genet.

[14] Lowe GC, Luheshi GN, Williams S (2008). Maternal infection and fever during late gestation are associated with altered synaptic transmission in the hippocampus of juvenile offspring rats. Am J Physiol.

[15] Mariani J, Coppola G, Zhang P, Abyzov A, Provini L, Tomasini L (2015). FOXG1-dependent dysregulation of GABA/glutamate neuron differentiation in autism spectrum disorders. Cell.

[16] Martynoga B, Morrison H, Price DJ, Mason JO (2005). Foxg1 is required for specification of ventral telencephalon and region-specific regulation of dorsal telencephalic precursor proliferation and apoptosis. Dev Biol.

[17] Matcovitch-Natan O, Winter DR, Giladi A, Vargas Aguilar S, Spinrad A, Sarrazin S (2016). Microglia development follows a stepwise program to regulate brain homeostasis. Science.

[18] Matsuhashi S, Kamiryo T, Blumberg PM, Linnett P, Willoughby E, Strominger JL (1974). Mechanism of action and development of resistance to a new amidino penicillin. J Bacteriol.

[19] O’Neill LA, Golenbock D, Bowie AG (2013). The history of Toll-like receptors - redefining innate immunity. Nat Rev Immunol.

[20] Orihuela CJ, Fillon S, Smith-Sielicki SH, El Kasmi KC, Gao G, Soulis K (2006). Cell wall-mediated neuronal damage in early sepsis. Infect Immun.

[21] Prinz M, Priller J (2014). Microglia and brain macrophages in the molecular age: from origin to neuropsychiatric disease. Nat Rev Neurosci.

[22] Robbins JR, Bakardjiev AI (2012). Pathogens and the placental fortress. Curr Opin Microbiol.

[23] Seoane J, Le HV, Shen L, Anderson SA, Massague J (2004). Integration of Smad and forkhead pathways in the control of neuroepithelial and glioblastoma cell proliferation. Cell.

[24] Sorensen HJ, Mortensen EL, Reinisch JM, Mednick SA (2009). Association between prenatal exposure to bacterial infection and risk of schizophrenia. Schizophrenia Bull.

[25] Tuomanen E, Liu H, Hengstler B, Zak O, Tomasz A (1985). The induction of meningeal inflammation by components of the pneumococcal cell wall. J Infect Dis.

[26] Tuomanen E, Tomasz A, Hengstler B, Zak O (1985). The relative role of bacterial cell wall and capsule in the induction of inflammation in pneumococcal meningitis. J Infect Dis.

[27] Yoshimura A, Lien E, Ingalls RR, Tuomanen E, Dziarski R, Golenbock D (1999). Cutting edge: recognition of Gram-positive bacterial cell wall components by the innate immune system occurs via Toll-like receptor 2. J Immunol.

